# Regional and epidemiological characteristics of tuberculosis and treatment outcomes in West China

**DOI:** 10.3389/fpubh.2023.1254579

**Published:** 2023-11-08

**Authors:** Qiaolan Wang, Jiangchuan Zhu, Luoning Zhang, Linshen Xie

**Affiliations:** ^1^West China School of Public Health and West China Fourth Hospital, Sichuan University, Chengdu, Sichuan, China; ^2^Dazhu County Center for Disease Control and Prevention, Dazhou, Sichuan, China

**Keywords:** tuberculosis, epidemic status, treatment outcome, population, temporal variation

## Abstract

**Objective:**

To understand the prevalence and treatment outcome of tuberculosis in a typically regional County from 2016 to 2021, so as to provide reference and basis for the prevention and treatment of tuberculosis in this area.

**Methods:**

Descriptive epidemiological methods were used to analyze the population, time and location distribution of pulmonary tuberculosis in Dazhu County from 2016 to 2021. The incidence rates were compared by Chi-square test and trend test, time distribution combined with seasonal index analysis, and the test level was *α* = 0.05.

**Results:**

A total of 2,899 cases of pulmonary tuberculosis were reported in Dazhu County from 2016 to 2021, with an average annual incidence rate of 44.29/100,000 and standardized reported incidence rate was 36.77/100,000, showing a downward trend year by year (*χ*^2^ trend = 124.629, *p* < 0.001). A total of 955 cases of pathogen positive were reported, showing an increasing trend year by year (*χ*^2^ trend = 59.773, *p* < 0.001). In terms of time distribution, the incidence rate was high in autumn and winter, and September and December were the peak of the disease in the whole year, and the overall trend increased first, then decreased and once again increased (*F* = 5.861, *p* < 0.05). In regional distribution, the highest annual average reported incidence rate was in concentrated population. The incidence rate of male was higher than female in population distribution. After standardization, the overall incidence rate increased from 34 to 45 years old (*χ*^2^ trend = 6963.101, *p* < 0.001), and decreased after 45 years old (*χ*^2^ trend = 1104.393, *p* < 0.001). The occupation distribution is mainly farmers (82.75%). The overall arrival rate and cure rate of patients showed an upward trend year by year (*χ*^2^ trend = 4.306, *χ*^2^ trend = 5.772, *p* < 0.001).

**Conclusion:**

The incidence rate of pulmonary tuberculosis in this regional county is decreasing year by year. Male patients are higher than female patients and have certain seasonal characteristics. Attention should be paid to male, older adult, farmers and other groups, and corresponding measures should be taken to strengthen the prevention and treatment of tuberculosis in high incidence areas.

## 1. Introduction

Tuberculosis is a chronic infectious disease caused by *Mycobacterium tuberculosis*, with fever, chest pain, cough, hemoptysis, emaciation and fatigue as the main manifestations, involving many organs of the body, of which pulmonary tuberculosis is the most common, accounting for 80–90% of the total tuberculosis in various organs. Droplet transmission is its main route of transmission, sputum excretion is called infectious pulmonary tuberculosis, except a few can be acute, the clinical process is mostly chronic (1). With the continuous improvement of living standards and medical conditions, although tuberculosis has been gradually brought under control, the current situation is still not optimistic. According to《Global tuberculosis report2020》 (2) released by the World Health Organization (WHO), tuberculosis is the second most fatal infectious disease after COVID-19, ranking the 13th cause of death in the world, and China is still on the global list of countries with high TB burden. The report also shows that the number of deaths from tuberculosis increased in 2019–2021, and there was a destructive change in the downward trend in 2005–2019. At the same time, the number of tuberculosis cases in 2020–2021 also showed an upward trend, reversing the downward trend in the past two decades. Therefore, tuberculosis is still a major infectious disease harmful to health and has become a public health problem concerned by many countries. Ending the TB epidemic by 2030 is one of the health-related targets of the United Nations sustainable development goals. As one of the countries with high TB burden, China has been committed to the prevention and control of tuberculosis. The incidence of tuberculosis in China has been on a downward trend from 2000 to 2021, with the number of cases falling from the second in 2020 to the third in 2021 (3). The overall treatment coverage rate is also at a high level. However, some work is still weak, such as the low reporting rate of children, which may be related to the gap in the supervision of tuberculosis in children in addition to the incidence of children themselves.

The effective prevention and treatment of tuberculosis depend on the principles of “early detection, early diagnosis, and early treatment.” These principles play a vital role in reducing the morbidity and mortality associated with tuberculosis (4). It is essential to conduct epidemiological studies to understand the disease’s characteristics, identify high-risk groups and tuberculosis patients promptly, and implement effective measures to reduce the incidence and the disease burden of tuberculosis. This study was carried out in representative areas of China to assess the current epidemiological situation, analyze key points for prevention and control, and highlight previously overlooked aspects. The findings serve as a valuable resource for future efforts in tuberculosis prevention and treatment, guiding the rational allocation of health resources and the formulation of health policies in the region.

## 2. Materials and methods

### 2.1. Source of information

The data for this study were collected from pulmonary tuberculosis patients registered in Dazhu County, Sichuan Province, from 2016 to 2021 through the Tuberculosis Information Management system, a subsystem of the “China Disease Prevention and Control Information system.” The data collection process involved four investigators: one for initial screening based on the research timeline and two others for a secondary screening based on inclusion and exclusion criteria. The final investigator conducted quality control and a comprehensive review of the database.

The collected data encompassed various parameters, including gender, age, occupation, area, patient source, initial diagnosis time, treatment classification, laboratory etiological diagnosis results, drug sensitivity test results, the number of patients in place, and patient treatment outcomes. Demographic data, such as gender and age-specific registered population figures, were obtained from the outpatient center of the study area.

The study area, Dazhu County in Sichuan Province, is a region with diverse characteristics, comprising urban areas, villages, and towns, with a sizable resident population engaged in various occupations. These features contribute to the area’s representative significance for the study.

### 2.2. Selection criteria

1. Inclusion criteria: ① Diagnostic criteria of pulmonary tuberculosis: accorded with 《Diagnosis of Pulmonary Tuberculosis》(WS288-2017); ② Complete case information; ③《WS196-2017 Tuberculosis Classification》 included tuberculous pleurisy in the classification of pulmonary tuberculosis, so the subjects included in this study were patients with tuberculous pleurisy.
2. Exclusion criteria: ① Patients with non-tuberculosis; ② Patients with non-pulmonary tuberculosis.

### 2.3. Partial index definition

1. Incidence rate = (Register patients/The population of the area at the end of the year)*100%
2. Overall arrival rate = (The actual number of referrals+The number of tracked digits+The number of other methods/The actual number of patients with pulmonary tuberculosis who should be referred)*100%
3. Cure rate = (Number of cured patients/Number of patients with sputum-coated positive patients)*100%

### 2.4. Research methods

Through the description of epidemiological methods, the incidence trend, regional, population distribution and treatment outcome of reported patients with pulmonary tuberculosis from 2016 to 2021. The study population was divided into 9 age groups: 0–4 years old, 5–14 years old, 15–24 years old, 25–34 years old, 35–44 years old, 45–54 years old, 55–64 years old, 65–74 years old and over 75.

### 2.5. Statistical analysis

Pulmonary tuberculosis database was established by Excel2021 and statistical analysis was carried out by SPSS26.0. The incidence rate was tested by chi-square test, the incidence trend was tested by chi-square trend test, and the law of seasonal change was analyzed by curvilinear regression combined with seasonal index. The test level is *α* = 0.05 (take the composition of Chinese census data as the standard population to calculate the standardized incidence rate).

## 3. Results

### 3.1. Epidemic overview

The total number of TB patients included in the categorized management in Dazhu County from 2016–2021 was 2,899, with an average annual reported incidence rate of 44.29/100,000 and a standardized average annual reported incidence rate of 36.77/100,000. The difference in the standardized incidence rate between years was statistically significant (*χ*^2^ = 128.596, *p* < 0.001), and the reported incidence rate decreased from 52.42/100,000 (standardized incidence rate of 51.62/100,000) in 2016 to 34.18/100,000 (standardized incidence rate of 26.55/100,000) in 2021, and the overall incidence rate and standardized incidence rate of pulmonary TB in the past 6 years were decreasing trend year by year (*χ*^2^ trend = 72.524, *p* < 0.001, *χ*^2^ trend = 124.629, *p* < 0.001). The pathogenic positive incidence rate was 14.59/100,000, and its reported incidence rate showed an increasing trend year by year (*χ*^2^ tren*d* = 59.773, *p* < 0.001). In this area, a total of 53 people were resistant to drugs from 2016 to 2021, including 18 with single drug resistance, 30 with multi-drug resistance and 5 with poly-resistance. The total drug resistance rate in 6 years was 1.82% (Table 1).

**Table 1:** Incidence of pulmonary tuberculosis.

Years	Gender	Number of population	Register	Incidence rate	Standardized incidence rate	Pathogenic positive patients	Pathogenic positive incidence rate	Number of patients with drug resistance
			patients		(1/100,000)		(1/100,000)	
2016	Male	591,446	430	72.7	71.06	91	15.39	20
	Female	522,737	154	29.46	30.51	20	3.83	
	Subtotal	1,114,183	584	52.42	51.62	111	9.96	
2017	Male	590,444	445	75.37	60.85	98	16.6	12
	Female	522,623	128	24.49	22.23	37	7.08	
	Subtotal	1,113,067	573	51.48	42.8	135	12.13	
2018	Male	583,941	405	69.36	56.29	108	18.5	3
	Female	516,174	134	25.96	24.88	29	5.62	
	Subtotal	1,100,115	539	48.99	41.29	137	12.45	
2019	Male	574,811	339	58.98	46.85	124	21.57	6
	Female	507,442	109	21.48	19.3	26	5.12	
	Subtotal	1,082,253	448	41.4	33.87	150	13.86	
2020	Male	569,134	295	51.83	39.31	164	28.82	4
	Female	502,423	96	19.11	18.05	38	7.56	
	Subtotal	1,071,557	391	36.49	29.14	202	18.85	
2021	Male	565,812	258	45.6	33.28	152	26.86	8
	Female	499,209	106	21.23	19.41	68	13.62	
	Subtotal	1,065,021	364	34.18	26.55	220	20.66	
Summation	Male	3,475,588	2,172	62.49	50.13	737	21.21	53
	Female	3,070,608	727	23.68	21.86	218	7.1	
	Subtotal	6,546,196	2,899	44.29	36.77	955	14.59	

### 3.2. Time variation distribution

**Figure 1:**
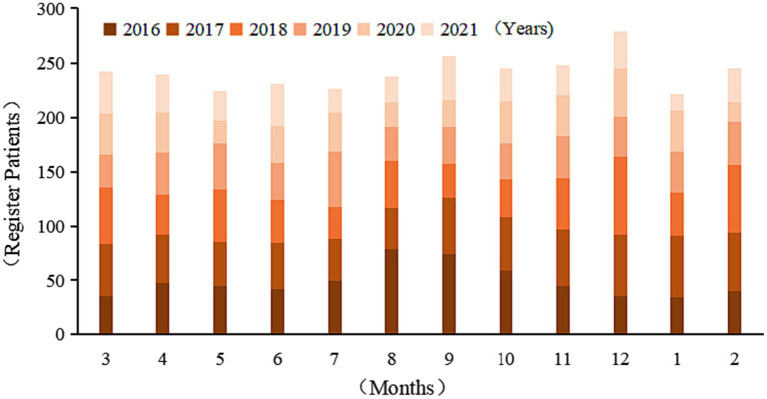
Time distribution of pulmonary tuberculosis patients in Dazhu County from 2016 to 2021.

Patients were reported cumulatively in all months, with a high prevalence in autumn and winter. Combining the cumulative number of reported cases over the years and the seasonal index, a comprehensive analysis showed that September and December were the two peak points of the disease throughout the year, with December being the highest peak (9.62% of the total). The overall trend was up, then down and then up month by month throughout the year (curve regression Cubic *F* = 5.861, *p* = 0.02 < 0.05) (Table 2; Figure 1).

**Table 2:** Distribution of tuberculosis patients in different months.

Quarter	Month	The number of reported cases						Summation	Proportion (%)	Summation	Seasonal index (fi)
		2016	2017	2018	2019	2020	2021				
Spring	3	35	48	53	30	38	39	243	8.38	24.42	1.01
	4	47	45	37	39	37	35	240	8.28		0.99
	5	45	40	49	42	21	28	225	7.76		0.93
Summer	6	42	42	40	34	34	39	231	7.97	23.97	0.96
	7	49	39	30	51	36	21	226	7.80		0.94
	8	79	38	43	31	23	24	238	8.21		0.99
Autumn	9	74	52	31	34	25	41	257	8.87	25.87	1.06
	10	59	49	35	33	39	30	245	8.45		1.01
	11	45	52	47	39	38	27	248	8.55		1.03
Winter	12	35	57	72	37	44	34	279	9.62	25.73	1.15
	1	34	57	40	38	38	15	222	7.66		0.92
	2	40	54	62	40	18	31	245	8.45		1.01
Summation		584	573	539	448	391	364	2,899	100.0	100	1.00

### 3.3. Regional distribution

There are 31 streets and towns under the jurisdiction of Dazhu County, including 3 urban streets (population concentrated areas) and 28 surrounding towns. The highest average annual incidence of tuberculosis in urban areas is 33.05/100,000 in Zhuyang Street, and the highest average annual incidence of tuberculosis in towns is 106.90/100,000 in Zhonghua Town.

### 3.4. Population distribution

#### 3.4.1. Gender distribution

The number of registered incidences was higher in men than in women, with a sex ratio of 2.99:1, and the average annual incidence rate was higher in men (62.49/100,000, with a standardized incidence rate of 50.13/100,000) than in women (23.68/100,000, with a standardized incidence rate of 21.86/100,000) (*χ*^2^ = 554.931, *p* < 0.001; *χ*^2^ = 353.562, *p* < 0.001).

#### 3.4.2. Age distribution

The age of TB patients was organized into nine groups for analysis in association with the actual situation in the region. After standardization, the overall incidence rate increased from 34 to 45 years old (*χ*^2^ trend = 6963.101, *p* < 0.001), and decreased after 45 years old (*χ*^2^ trend = 1104.393, *p* < 0.001) (Table 3).

**Table 3:** Age-specific incidence of tuberculosis.

Age	Number of population	Number of patients	The reported incidence (1/10,0000)	Standardized incidence (1/10,0000)
0–4	301,797	0	0.00	0.00
5–14	788,629	12	1.52	26.40
15–24	815,761	312	38.25	608.12
25–34	884,362	315	35.62	702.05
35–44	791,547	320	40.43	619.34
45–54	1,298,072	762	58.70	702.67
55–64	759,667	576	75.82	537.58
65–74	590,094	468	79.31	385.44
75-	316,267	134	42.37	94.91
Summation	6,549,196	2,899	44.29	36.77

#### 3.4.3. Occupational distribution

**Figure 2:**
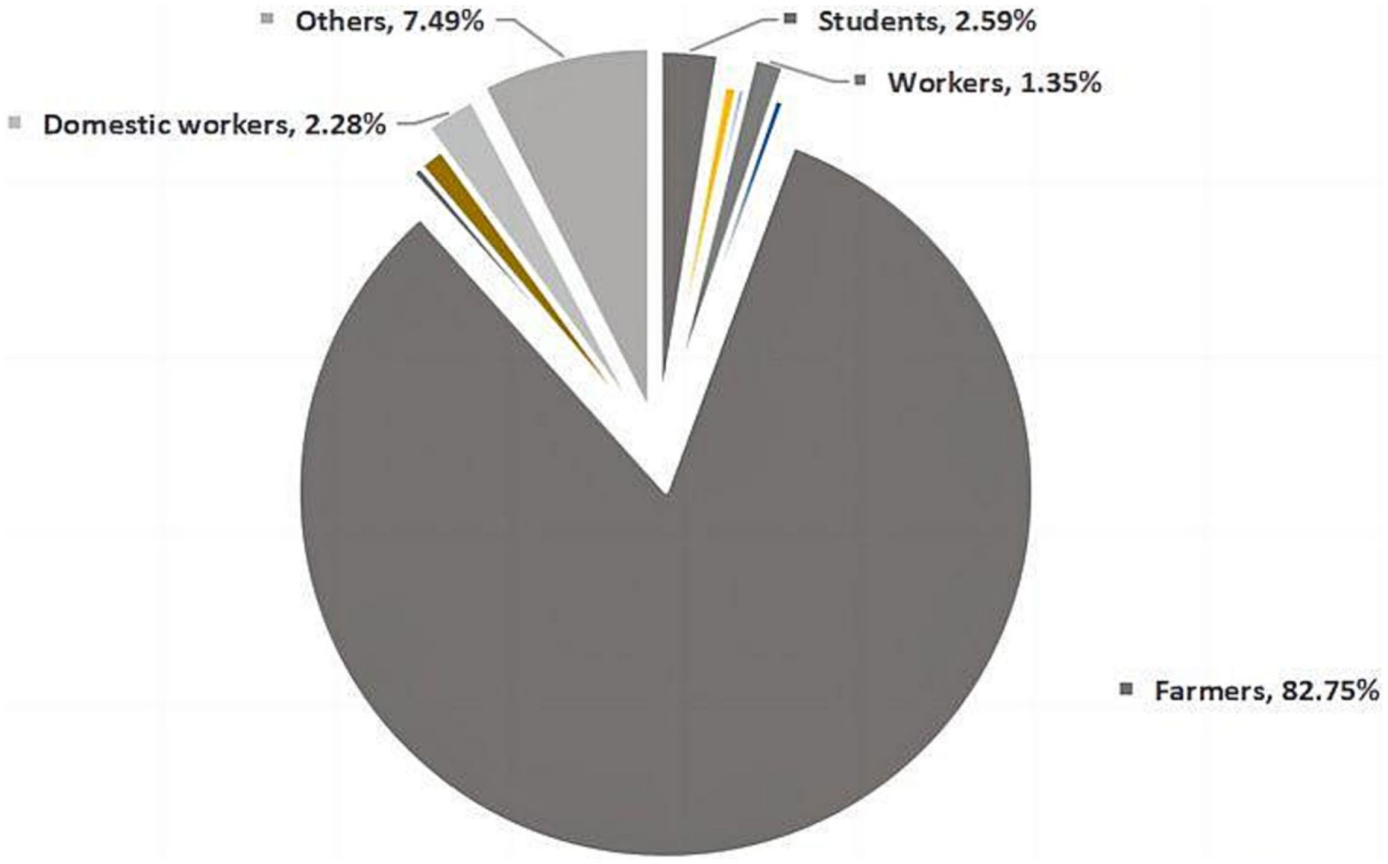
Occupational distribution figure.

The survey showed that farmers accounted for the highest proportion of TB patients, 82.75%. In the past 6 years, the proportion of farmers are in the first place of each occupation. Students, domestic workers, and workers accounted for 2.59, 2.28, and 1.35%, respectively (Figure 2).

### 3.5. Treatment regression status

#### 3.5.1. Overall rate of suspected cases

A total of 2,704 suspected TB cases were reported by medical institutions over the past 6 years, and 2,683 TB cases were referred into place, with an overall arrival rate of 99.22%; the highest overall arrival rate of 99.74% was achieved in 2021, and the lowest overall arrival rate of 98.73% was achieved in 2016. The overall rate was increasing year by year (*χ*^2^ trend = 4.306, *p* = 0.03 < 0.05) (Table 4).

**Table 4:** Tracking status of suspected tuberculosis patients in Dazhu County from 2016 to 2021.

Years	Number of reported patients	Referral tracking				Overall arrival rate
		Referral	Tracking	Other	Total	
2016	631	71	546	6	623	98.73
2017	370	61	297	6	364	98.38
2018	452	66	383	1	450	99.56
2019	469	63	401	3	467	99.57
2020	390	150	235	3	388	99.49
2021	392	153	236	2	391	99.74
Total	2,704	564	2,098	21	2,683	99.23

#### 3.5.2. Treatment regression status

Over the 6 years, 900 of the registered patients were cured, 1,949 completed the course of treatment, and 7 patients died. The highest cure rate was 98.64% in 2021 and the lowest was 94.60% in 2016. The cure rate had a slight decrease in 2018, but still showed an overall upward trend (*χ*^2^ trend = 5.772, *p* = 0.02 < 0.05). (Sputum-coated positive patients with a full course of treatment and final sputum negativity were judged as cured, and the cure rate was calculated only for sputum-coated positive patients) (Table 5).

**Table 5:** Treatment regression status of tuberculosis patients in Dazhu County from 2016 to 2021.

Treatment regression status	Number of cases						Total	Proportion (%)
	2016	2017	2018	2019	2020	2021		
Cure	105	127	118	143	190	217	900	30.87
Complete the course of treatment	470	440	416	296	202	130	1949	67.4
Non-tuberculosis death	1	4	2	3	4	9	23	0.79
Fail	2	0	0	1	0	0	3	0.1
Lose	0	0	0	0	0	0	0	0
Diagnostic change	2	0	2	4	1	2	11	0.38
Adverse reaction	0	0	0	0	0	0	0	0
Transferred to multi-drug resistance treatment	0	0	0	0	0	6	6	0.21
Other	0	0	0	0	0	0	0	0
Refuse treatment	0	0	0	0	0	0	0	0
Total	584	573	539	448	391	364	2,899	100

## 4. Discussion

The study found that the average annual incidence of TB incidence in this locality from 2016 to 2021 showed a decreasing trend, while lower than the national (5–8) and provincial (9–14) incidence levels in the same period. It is lower than the average annual incidence of tuberculosis in Bazhong City from 2006 to 2015 (96.15/100000) (15) and Luzhou City from 2008 to 2017 (82.29/100000) (16) of the same province, implying that the TB epidemic in this area has been controlled to some extent in recent years, and the implementation of the action plan to curb TB (17) has been effective, which is inextricably linked to the active promotion of prevention and treatment by medical institutions and the continuous improvement of TB publicity and education. The report showed that the spread of TB can be controlled by enhancing the knowledge and policy of TB education (18); the area is included remote towns, where some of the patients may not immediately go to the medical institutions for treatment due to unfavorable transportation, lack of medical resources, and weak awareness of prevention (19, 20), resulting in underreporting of patients and possibly causing the number of registered incidences lower than the actual number of incidence, suggesting that the administration should increase economic investment in remote townships to solve the problem, and timely detection of TB patients and active treatment to reduce the chances of transmission. The pathogenic positivity rate in the area is increasing year by year, implying that the transmission of TB may still be caused within a certain period of time, and attention should be paid to prevention.

Analysis of the time and regional distribution shows that the incidence of the disease occurs throughout the year, but it is high in winter, which is consistent with the pattern of respiratory diseases being high in winter, when the weather is cold and the temperature is low, the climate is dry, the living environment is relatively poor, and the immunity of the organism decreases compared with other seasons (21). On the other hand, some patients are unwilling to treat or do not standardize their treatment when they are working outside, and only after they return home during the Spring Festival do they begin to register and standardize their treatment, which also contributes to the higher incidence of TB in winter than in other seasons in this region. It is suggested that in winter, we should advocate residents to improve their immunity, indoor ventilation, avoid crowded places as much as possible, and carry out health education activities for workers to encourage them to seek medical treatment as soon as possible; the highest average annual incidence rate in Zhuyang Street and Zhonghua Town. In addition to better medical conditions in urban areas, it easier to find TB patients (22). The more direct reason is that there is a large flow of people and many migrant workers in this area. Therefore, we should pay attention to the urban areas with high incidence, and formulate reasonable measures according to the actual situation.

The population distribution showed that the incidence of TB was higher in males than in females, consistent with the epidemiological characteristics of TB in China and abroad as well as most studies (23, 24), with a large range of social activities and a higher proportion of bad behavioral habits and lifestyles such as long-term smoking and alcohol consumption in males. Among the reported cases, the population with TB incidence is mainly concentrated around 15–54 years of age, related to more opportunities for young adults to go out, a wider range of daily life activities, and a wider variety of people in contact; the incidence is also relatively high among the older adult around 70 years of age, related to their own reduced organism immunity, more chronic underlying diseases, and poor hygiene habits (25, 26); the occupational distribution is mainly dominated by farmers, accounting for above 80%. This is because of the large base of farmers and the poor economic conditions, the high labor intensity, the low standard of living, the relatively low level of education of most of them, and the lower knowledge of TB prevention and treatment (27), which not only increase the chance of TB transmission but also increase the difficulty of TB prevention and treatment. In addition to focusing on farmers, it is also important to increase the prevention of students, who spend most of their time in a more intensive environment, coupled with the high level of stress among students and the relatively lower immunity of the body (28, 29), which is also prone to TB. Domestic workers and workers account for a larger proportion of the population than other occupations. There is a relationship between the nature of work, the high number of contacts, and the confined working environment.

The tuberculosis incidence rate in Dazhu County from 2016 to 2021 has gradually declined, while the overall arrival rate and cure rate have increased each year. This indicates successful tuberculosis prevention and control efforts in the region. Various strategies have been employed to identify tuberculosis patients and provide prompt diagnosis and treatment in designated medical facilities. These measures have improved the quality of care for tuberculosis patients. However, future efforts should focus on strengthening surveillance, risk assessment, and health education for high-risk groups. Increased economic investment is also needed, particularly in remote areas, to address transportation and resource challenges. This will help prevent tuberculosis patients from going outside the management system and potentially spreading the disease.

## Data availability statement

The original contributions presented in the study are included in the article/supplementary material, further inquiries can be directed to the corresponding author.

## Author contributions

QW: Conceptualization, Data curation, Investigation, Methodology, Project administration, Writing – original draft, Writing – review & editing. JZ: Investigation, Methodology, Project administration, Writing – review & editing. LZ: Data curation, Investigation, Writing – original draft. LX: Conceptualization, Data curation, Formal analysis, Investigation, Methodology, Project administration, Resources, Writing – review & editing.
